# Supplementary material for: Asymmetric biparental and inefficient horizontal transmission of paralysis-causing sigmavirus in Queensland fruit fly
Source: Heredity (Edinb). 2026 Apr 6;135(5):372–83. doi: 10.1038/s41437-026-00839-4 (PMC13219430; doi:10.1038/s41437-026-00839-4)
Supplement: Supplementary file 1 — Supplementary Information 1-10 [file 41437_2026_839_MOESM1_ESM.pdf]

Supplementary Information (SI)

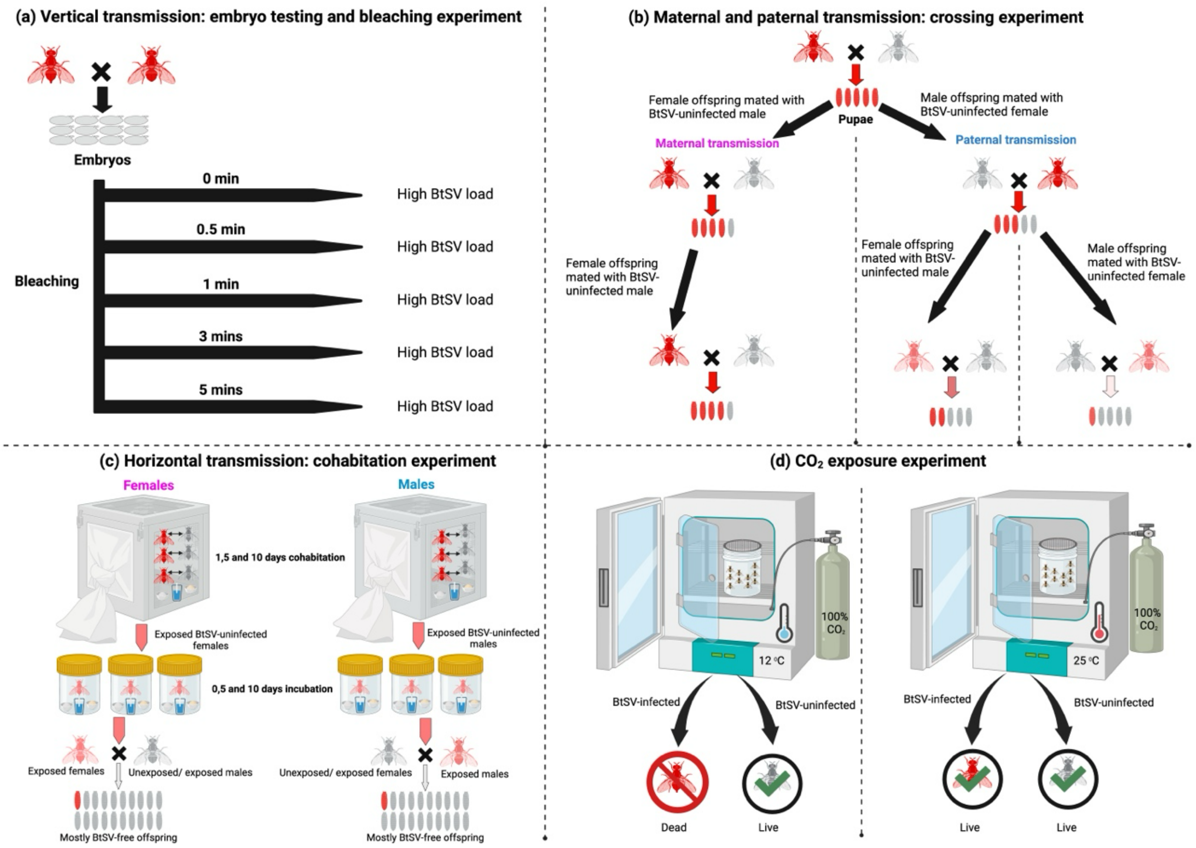

SI 1. Overview of the experiments performed to assess transmission pathways and host effects of *Sigmavirus tryoni* (BtSV) in *Bactrocera tryoni*. Experiments to assess **(a)** vertical transmission of BtSV within embryos (embryo testing and bleaching experiments), **(b)** maternal and paternal BtSV transmission (crossing experiments), **(c)** horizontal BtSV transmission (cohabitation experiments) and **(d)** CO_2-triggered paralysis (CO_2 exposure experiment).

SI 2. Nucleotide sequence of *Sigmavirus tryoni* variant (BtSV1) found in C28+ and LE+

>C28+.Female1.SV1
GCTATCGGGTGGTTTGTGGAGTCTGACTCGTGTTTGGCTCATGCCATTTATGATAACTTAAAGGCCCTAACAGGTGAGGA
CTGGAGTAATTCAGTTGAAGGATTCAAACGAACTGGGTCAGCATTACACAGATTTTCTTGTTCGAGGCAAAGTGCTGGAG
GGTTTATTGCATCTAGTCCAATCAAGTCGACGTATATGATATCAACAACAAACACATTATCTGAGCTCGGTGATAAAAACT
ATGATTTTATTTTCCAAACATTGTTGCTCTACACTGAAATCACTTGCGGAGAATTACATGCTAATAACCCGAACTCAGGATA
TTACCATATGCATATCTCATGTTCAAATTGTTTACGAGAAATTGAGGAACCAATATTGAATACCTCTGTGACGCCAACCTAT
CGT
>C28+.Male1.SV1
GCTATCGGGTGGTTTGTGGAGTCTGACTCGTGTTTGGCTCATGCCATTTATGATAACTTAAAGGCCCTAACAGGTGAGGA
CTGGAGTAATTCAGTTGAAGGATTCAAACGAACTGGGTCAGCATTACACAGATTTTCTTGTTCGAGGCAAAGTGCTGGAG
GGTTTATTGCATCTAGTCCAATCAAGTCGACGTATATGATATCAACAACAAACACATTATCTGAGCTCGGTGATAAAAACT
ATGATTTTATTTTCCAAACATTGTTGCTCTACACTGAAATCACTTGCGGAGAATTACATGCTAATAACCCGAACTCAGGATA
TTACCATATGCATATCTCATGTTCAAATTGTTTACGAGAAATTGAGGAACCAATATTGAATACCTCTGTGACGCCAACCTAT
CGT
>C28+.Male2.SV1
GCTATCGGGTGGTTTGTGGAGTCTGACTCGTGTTTGGCTCATGCCATTTATGATAACTTAAAGGCCCTAACAGGTGAGGA
CTGGAGTAATTCAGTTGAAGGATTCAAACGAACTGGGTCAGCATTACACAGATTTTCTTGTTCGAGGCAAAGTGCTGGAG
GGTTTATTGCATCTAGTCCAATCAAGTCGACGTATATGATATCAACAACAAACACATTATCTGAGCTCGGTGATAAAAACT
ATGATTTTATTTTCCAAACATTGTTGCTCTACACTGAAATCACTTGCGGAGAATTACATGCTAATAACCCGAACTCAGGATA
TTACCATATGCATATCTCATGTTCAAATTGTTTACGAGAAATTGAGGAACCAATATTGAATACCTCTGTGACGCCAACCTAT
CGT
>LE+.Female1.SV1
GCTATCGGGTGGTTTGTGGAGTCTGACTCGTGTTTGGCTCATGCCATTTATGATAACTTAAAGGCCCTAACAGGTGAGGA
CTGGAGTAATTCAGTTGAAGGATTCAAACGAACTGGGTCAGCATTACACAGATTTTCTTGTTCGAGGCAAAGTGCTGGAG
GGTTTATTGCATCTAGTCCAATCAAGTCGACGTATATGATATCAACAACAAACACATTATCTGAGCTCGGTGATAAAAACT
ATGATTTTATTTTCCAAACATTGTTGCTCTACACTGAAATCACTTGCGGAGAATTACATGCTAATAACCCGAACTCAGGATA
TTACCATATGCATATCTCATGTTCAAATTGTTTACGAGAAATTGAGGAACCAATATTGAATACCTCTGTGACGCCAACCTAT
CGT
>LE+.Male1.SV1
GCTATCGGGTGGTTTGTGGAGTCTGACTCGTGTTTGGCTCATGCCATTTATGATAACTTAAAGGCCCTAACAGGTGAGGA
CTGGAGTAATTCAGTTGAAGGATTCAAACGAACTGGGTCAGCATTACACAGATTTTCTTGTTCGAGGCAAAGTGCTGGAG
GGTTTATTGCATCTAGTCCAATCAAGTCGACGTATATGATATCAACAACAAACACATTATCTGAGCTCGGTGATAAAAACT
ATGATTTTATTTTCCAAACATTGTTGCTCTACACTGAAATCACTTGCGGAGAATTACATGCTAATAACCCGAACTCAGGATA
TTACCATATGCATATCTCATGTTCAAATTGTTTACGAGAAATTGAGGAACCAATATTGAATACCTCTGTGACGCCAACCTAT
CGT
>LE+.Male2.SV1
GCTATCGGGTGGTTTGTGGAGTCTGACTCGTGTTTGGCTCATGCCATTTATGATAACTTAAAGGCCCTAACAGGTGAGGA
CTGGAGTAATTCAGTTGAAGGATTCAAACGAACTGGGTCAGCATTACACAGATTTTCTTGTTCGAGGCAAAGTGCTGGAG
GGTTTATTGCATCTAGTCCAATCAAGTCGACGTATATGATATCAACAACAAACACATTATCTGAGCTCGGTGATAAAAACT
ATGATTTTATTTTCCAAACATTGTTGCTCTACACTGAAATCACTTGCGGAGAATTACATGCTAATAACCCGAACTCAGGATA
TTACCATATGCATATCTCATGTTCAAATTGTTTACGAGAAATTGAGGAACCAATATTGAATACCTCTGTGACGCCAACCTAT
CGT

SI 3. BtSV loads across developmental stages

Kruskal-Wallis rank sum test

Kruskal-Wallis chi-squared = 17.99, df = 4, p-value = 0.0012

Dunn (1964) Kruskal-Wallis multiple comparison
p-values adjusted with the Benjamini-Hochberg method.

	Comparison	Z	P.unadj	P.adj
1	Embryos - Female	-2.52435736	0.0115910033	0.038636678
2	Embryos - Larvae	-1.70576206	0.0880523901	0.176104780
3	Female - Larvae	1.13160847	0.2577990769	0.368284396
4	Embryos - Male	-3.06839989	0.0021520842	0.010760421
5	Female - Male	-0.47115466	0.6375302971	0.708366997
6	Larvae - Male	-1.67565101	0.0938065718	0.156344286
7	Embryos - Pupae	-3.71802824	0.0002007838	0.002007838
8	Female - Pupae	-0.51139998	0.6090710073	0.761338759
9	Larvae - Pupae	-2.01226618	0.0441918858	0.110479714
10	Male - Pupae	0.03264255	0.9739596363	0.973959636

SI 4. Test for transovarian or transovum transmission of BtSV in LE+ embryos

Kruskal-Wallis rank sum test

Kruskal-Wallis chi-squared = 9.539, df = 4, p-value = 0.04895

Dunn (1964) Kruskal-Wallis multiple comparison
p-values adjusted with the Benjamini-Hochberg method.

	Comparison	Z	P.unadj	P.adj
1	1min - 3min	1.10452021	0.269367543	0.44894591
2	1min - 5min	-0.44871133	0.653639908	0.72626656
3	3min - 5min	-1.55323154	0.120367829	0.24073566
4	1min - Female	-0.55677679	0.577679940	0.72209993
5	3min - Female	-1.88887828	0.058908140	0.19636047
6	5min - Female	-0.01561056	0.987545078	0.98754508
7	1min - 0min	1.72581282	0.084381092	0.21095273
8	3min - 0min	0.62129262	0.534407111	0.76343873
9	5min - 0min	2.17452416	0.029665793	0.14832897
10	Female - 0min	2.63818537	0.008335101	0.08335101

SI 5. Test for transovarian or transovum transmission of BtSV in embryos of ($LE^+ \times B6^-$) $\times B6^-$ and $B6^- \times$ ($LE^+ \times B6^-$) in two families

Embryos of ($LE^+ \times B6^-$) $\times B6^-$ family 5

Kruskal-Wallis rank sum test

Kruskal-Wallis chi-squared = 5.4218, df = 5, p-value = 0.3666

Dunn (1964) Kruskal-Wallis multiple comparison
p-values adjusted with the Benjamini-Hochberg method.

	Comparison	Z	P.unadj	P.adj
1	1min - 0.5min	-0.9341987	0.35020139	0.6566276
2	1min - 3min	-0.4670994	0.64042879	0.7389563
3	0.5min - 3min	0.4670994	0.64042879	0.8005360
4	1min - 5min	0.4670994	0.64042879	0.8733120
5	0.5min - 5min	1.4012981	0.16112495	0.6042186
6	3min - 5min	0.9341987	0.35020139	0.7504315
7	1min - Female	0.2412091	0.80939308	0.8093931
8	0.5min - Female	1.4472545	0.14782567	0.7391284
9	3min - Female	0.8442318	0.39853991	0.6642332
10	5min - Female	-0.3618136	0.71749132	0.7687407
11	1min - 0min	1.1677484	0.24290826	0.6072707
12	0.5min - 0min	2.1019471	0.03555791	0.5333686
13	3min - 0min	1.6348478	0.10208096	0.7656072
14	5min - 0min	0.7006490	0.48352206	0.7252831
15	Female - 0min	1.2663476	0.20538865	0.6161659

Kruskal-Wallis rank sum test when compared all embryos into one pool with females
Kruskal-Wallis chi-squared = 0.098182, df = 1, p-value = 0.754

Embryos of ($LE^+ \times B6^-$) $\times B6^-$ family 6

Kruskal-Wallis rank sum test

Kruskal-Wallis chi-squared = 2.1491, df = 5, p-value = 0.8282

Dunn (1964) Kruskal-Wallis multiple comparison
p-values adjusted with the Benjamini-Hochberg method.

	Comparison	Z	P.unadj	P.adj
1	1min - 0.5min	0.46709937	0.6404288	1.0000000
2	1min - 3min	-0.46709937	0.6404288	1.0000000
3	0.5min - 3min	-0.93419873	0.3502014	1.0000000
4	1min - 5min	0.23354968	0.8153346	0.8735728
5	0.5min - 5min	-0.23354968	0.8153346	0.9407707
6	3min - 5min	0.70064905	0.4835221	1.0000000
7	1min - Female	0.66332496	0.5071225	1.0000000
8	0.5min - Female	0.06030227	0.9519149	0.9519149
9	3min - Female	1.26634765	0.2053886	1.0000000
10	5min - Female	0.36181361	0.7174913	1.0000000
11	1min - 0min	0.70064905	0.4835221	1.0000000
12	0.5min - 0min	0.23354968	0.8153346	1.0000000
13	3min - 0min	1.16774842	0.2429083	1.0000000
14	5min - 0min	0.46709937	0.6404288	1.0000000
15	Female - 0min	0.24120908	0.8093931	1.0000000

Kruskal-Wallis rank sum test when compared all embryos into one pool with females
Kruskal-Wallis chi-squared = 0.53455, df = 1, p-value = 0.4647

Embryos of B6^{-} × (LE^{+} × B6^{-}) family 5

Kruskal-Wallis rank sum test

Kruskal-Wallis chi-squared = 7.3333, df = 5, p-value = 0.197

Dunn (1964) Kruskal-Wallis multiple comparison
p-values adjusted with the Benjamini-Hochberg method.

	Comparison	Z	P.unadj	P.adj
1	1min - 0.5min	-0.2581989	0.79625341	0.7962534
2	1min - 3min	-0.5163978	0.60557662	0.8257863
3	0.5min - 3min	-0.2581989	0.79625341	0.8531287
4	1min - 5min	0.2581989	0.79625341	0.9187539
5	0.5min - 5min	0.5163978	0.60557662	0.9083649
6	3min - 5min	0.7745967	0.43857803	0.8223338
7	1min - Male	-1.4696938	0.14164469	0.7082235
8	0.5min - Male	-1.1430952	0.25299906	0.9487465
9	3min - Male	-0.8164966	0.41421618	1.0000000
10	5min - Male	-1.7962925	0.07244801	0.5433601
11	1min - 0min	0.5163978	0.60557662	1.0000000
12	0.5min - 0min	0.7745967	0.43857803	0.9398101
13	3min - 0min	1.0327956	0.30169958	0.9050987
14	5min - 0min	0.2581989	0.79625341	0.9953168
15	Male - 0min	2.1228911	0.03376298	0.5064447

Kruskal-Wallis rank sum test when compared all embryos into one pool with males

Kruskal-Wallis chi-squared = 6, df = 1, p-value = 0.01431

Embryos of B6^{-} × (LE^{+} × B6^{-}) family 6

Kruskal-Wallis rank sum test
Kruskal-Wallis chi-squared = 7.9091, df = 5, p-value = 0.1613

Dunn (1964) Kruskal-Wallis multiple comparison
p-values adjusted with the Benjamini-Hochberg method.

	Comparison	Z	P.unadj	P.adj
1	1min - 0.5min	0.2335497	0.81533459	0.8153346
2	1min - 3min	-0.4670994	0.64042879	0.8733120
3	0.5min - 3min	-0.7006490	0.48352206	0.9066039
4	1min - 5min	-0.2335497	0.81533459	0.8735728
5	0.5min - 5min	-0.4670994	0.64042879	0.9606432
6	3min - 5min	0.2335497	0.81533459	0.9407707
7	1min - Male	-1.5075567	0.13166802	0.6583401
8	0.5min - Male	-1.8090681	0.07044043	0.5283032
9	3min - Male	-0.9045340	0.36571230	0.9142807
10	5min - Male	-1.2060454	0.22779999	0.8542500
11	1min - 0min	0.4670994	0.64042879	1.0000000
12	0.5min - 0min	0.2335497	0.81533459	1.0000000
13	3min - 0min	0.9341987	0.35020139	1.0000000
14	5min - 0min	0.7006490	0.48352206	1.0000000
15	Male - 0min	2.1105794	0.03480848	0.5221272

Kruskal-Wallis rank sum test when compared all embryos into one pool with males
Kruskal-Wallis chi-squared = 6.8182, df = 1, p-value = 0.009023

SI. 6. Horizontal transmission of BtSV

Horizontal transmission of BtSV in female

Kruskal-Wallis rank sum test

Kruskal-Wallis chi-squared = 31.263, df = 10, p-value = 0.0005308

Dunn (1964) Kruskal-Wallis multiple comparison
p-values adjusted with the Benjamini-Hochberg method.

	Comparison	Z	P.unadj	P.adj
1	1:0 - 1:10	2.629213943	8.558250e-03	0.0588379689
2	1:0 - 1:5	0.553518725	5.799083e-01	0.7248853279
3	1:10 - 1:5	-2.075695218	3.792215e-02	0.1390478778
4	1:0 - 10:0	0.805593257	4.204774e-01	0.6423960501
5	1:10 - 10:0	-1.471372809	1.411903e-01	0.2986718365
6	1:5 - 10:0	0.326231980	7.442488e-01	0.8709295018
7	1:0 - 10:10	0.572570414	5.669356e-01	0.7251501816
8	1:10 - 10:10	-1.704395652	8.830720e-02	0.2312807505
9	1:5 - 10:10	0.093209137	9.257374e-01	0.9791453434
10	10:0 - 10:10	-0.208421967	8.348995e-01	0.9003818199
11	1:0 - 10:5	-0.226365048	8.209175e-01	0.9030092615
12	1:10 - 10:5	-2.503331114	1.230304e-02	0.0751852336
13	1:5 - 10:5	-0.705726325	4.803583e-01	0.6443831482
14	10:0 - 10:5	-0.923011568	3.560012e-01	0.6118770227
15	10:10 - 10:5	-0.714589601	4.748627e-01	0.6529361744
16	1:0 - 5:0	-0.253696082	7.997304e-01	0.9163577053
17	1:10 - 5:0	-2.882910025	3.940200e-03	0.0361184971
18	1:5 - 5:0	-0.807214807	4.195427e-01	0.6592814428
19	10:0 - 5:0	-1.025300509	3.052214e-01	0.5595725950
20	10:10 - 5:0	-0.792277666	4.281988e-01	0.6365117165
21	10:5 - 5:0	0.006657796	9.946879e-01	0.9946878870
22	1:0 - 5:10	0.645771846	5.184271e-01	0.6788926623
23	1:10 - 5:10	-1.983442097	4.731807e-02	0.1445829879
24	1:5 - 5:10	0.092253121	9.264969e-01	0.9614590821
25	10:0 - 5:10	-0.246338434	8.054203e-01	0.9040431493
26	10:10 - 5:10	-0.013315591	9.893760e-01	1.0000000000
27	10:5 - 5:10	0.785619871	4.320902e-01	0.6253937388
28	5:0 - 5:10	0.899467928	3.684035e-01	0.6140057860
29	1:0 - 5:5	1.499113213	1.338443e-01	0.3067264392
30	1:10 - 5:5	-1.130100730	2.584338e-01	0.4901330353
31	1:5 - 5:5	0.945594488	3.443555e-01	0.6109532294
32	10:0 - 5:5	0.492676868	6.222409e-01	0.7605166857
33	10:10 - 5:5	0.725699711	4.680229e-01	0.6600322650
34	10:5 - 5:5	1.524635173	1.273501e-01	0.3045328939
35	5:0 - 5:5	1.752809295	7.963475e-02	0.2189955545
36	5:10 - 5:5	0.853341367	3.934700e-01	0.6364956303
37	1:0 - Infected	-1.641692317	1.006538e-01	0.2516344616
38	1:10 - Infected	-4.120538995	3.779871e-05	0.0010394645
39	1:5 - Infected	-2.163554776	3.049854e-02	0.1198156754
40	10:0 - Infected	-2.212209374	2.695220e-02	0.1140285446
41	10:10 - Infected	-1.989396919	4.665741e-02	0.1509504424
42	10:5 - Infected	-1.225468502	2.203988e-01	0.4329261175
43	5:0 - Infected	-1.402505357	1.607644e-01	0.3274830206
44	5:10 - Infected	-2.250531852	2.441520e-02	0.1119030179
45	5:5 - Infected	-3.055069809	2.250081e-03	0.0309386196
46	1:0 - Uninfected	2.897423858	3.762411e-03	0.0413865161
47	1:10 - Uninfected	0.418577180	6.755252e-01	0.8076931379
48	1:5 - Uninfected	2.375561399	1.752228e-02	0.0963725231
49	10:0 - Uninfected	1.774542052	7.597353e-02	0.2199233843
50	10:10 - Uninfected	1.997354507	4.578669e-02	0.1573917335
51	10:5 - Uninfected	2.761282924	5.757478e-03	0.0452373244
52	5:0 - Uninfected	3.136610818	1.709128e-03	0.0313340193
53	5:10 - Uninfected	2.288584323	2.210352e-02	0.1105175781
54	5:5 - Uninfected	1.484046366	1.377966e-01	0.3031525648
55	Infected - Uninfected	4.306183703	1.660951e-05	0.0009135233

Horizontal transmission of BtSV in male

Kruskal-Wallis rank sum test
Kruskal-Wallis chi-squared = 28.936, df = 10, p-value = 0.001276
Dunn (1964) Kruskal-Wallis multiple comparison
p-values adjusted with the Benjamini-Hochberg method.

	Comparison	Z	P.unadj	P.adj
1	1:0 - 1:10	1.7064938	8.791617e-02	0.268632751
2	1:0 - 1:5	-0.2201928	8.257210e-01	0.908293144
3	1:10 - 1:5	-1.9266866	5.401870e-02	0.198068567
4	1:0 - 10:0	-1.1284879	2.591139e-01	0.431856569
5	1:10 - 10:0	-2.8349817	4.582831e-03	0.063013928
6	1:5 - 10:0	-0.9082951	3.637223e-01	0.540668324
7	1:0 - 10:10	-0.8257228	4.089613e-01	0.591917732
8	1:10 - 10:10	-2.5322167	1.133439e-02	0.069265736
9	1:5 - 10:10	-0.6055301	5.448269e-01	0.713463733
10	10:0 - 10:10	0.3027650	7.620689e-01	0.855383494
11	1:0 - 10:5	-0.6605783	5.088828e-01	0.682647679
12	1:10 - 10:5	-2.3670721	1.792944e-02	0.082176599
13	1:5 - 10:5	-0.4403855	6.596579e-01	0.788721427
14	10:0 - 10:5	0.4679096	6.398492e-01	0.782037957
15	10:10 - 10:5	0.1651446	8.688302e-01	0.918955016
16	1:0 - 5:0	-0.9908674	3.217503e-01	0.491563017
17	1:10 - 5:0	-2.6973612	6.989141e-03	0.054914679
18	1:5 - 5:0	-0.7706746	4.408998e-01	0.621781783
19	10:0 - 5:0	0.1376205	8.905404e-01	0.907031865
20	10:10 - 5:0	-0.1651446	8.688302e-01	0.936973742
21	10:5 - 5:0	-0.3302891	7.411815e-01	0.849270475
22	1:0 - 5:10	0.3578132	7.204831e-01	0.843118514
23	1:10 - 5:10	-1.3486806	1.774396e-01	0.375352948
24	1:5 - 5:10	0.5780060	5.632601e-01	0.720448945
25	10:0 - 5:10	1.4863011	1.371995e-01	0.342998751
26	10:10 - 5:10	1.1835360	2.365968e-01	0.419768461
27	10:5 - 5:10	1.0183915	3.084919e-01	0.484773062
28	5:0 - 5:10	1.3486806	1.774396e-01	0.390367066
29	1:0 - 5:5	0.4679096	6.398492e-01	0.799811547
30	1:10 - 5:5	-1.2385842	2.154995e-01	0.408705965
31	1:5 - 5:5	0.6881024	4.913883e-01	0.675658952
32	10:0 - 5:5	1.5963975	1.104001e-01	0.319579188
33	10:10 - 5:5	1.2936324	1.957924e-01	0.398836405
34	10:5 - 5:5	1.1284879	2.591139e-01	0.445352087
35	5:0 - 5:5	1.4587770	1.446265e-01	0.345845974
36	5:10 - 5:5	0.1100964	9.123329e-01	0.912332942
37	1:0 - Infected	-2.3945962	1.663868e-02	0.083193393
38	1:10 - Infected	-4.1010900	4.112085e-05	0.002261647
39	1:5 - Infected	-2.1744034	2.967485e-02	0.116579769
40	10:0 - Infected	-1.2661083	2.054743e-01	0.403610234
41	10:10 - Infected	-1.5688734	1.166774e-01	0.305583795
42	10:5 - Infected	-1.7340179	8.291490e-02	0.268254094
43	5:0 - Infected	-1.4037288	1.603996e-01	0.367582469
44	5:10 - Infected	-2.7524094	5.915851e-03	0.065074362
45	5:5 - Infected	-2.8625058	4.203056e-03	0.077056023
46	1:0 - Uninfected	1.5688734	1.166774e-01	0.320862985
47	1:10 - Uninfected	-0.1376205	8.905404e-01	0.924145673
48	1:5 - Uninfected	1.7890661	7.360417e-02	0.253014327
49	10:0 - Uninfected	2.6973612	6.989141e-03	0.064067125
50	10:10 - Uninfected	2.3945962	1.663868e-02	0.091512732
51	10:5 - Uninfected	2.2294516	2.578387e-02	0.109085606
52	5:0 - Uninfected	2.5597408	1.047503e-02	0.072015812
53	5:10 - Uninfected	1.2110601	2.258724e-01	0.414099319
54	5:5 - Uninfected	1.1009638	2.709124e-01	0.438240692
55	Infected - Uninfected	3.9634696	7.386829e-05	0.002031378

Comparison of acquired BtSV load between females and males of EC16$^-$

Kruskal-Wallis rank sum test
Kruskal-Wallis chi-squared = 1.141, df = 1, p-value = 0.2854

SI 7. Body region and tissue localisation of BtSV in LE+ females and males and in female and male offspring of LE+ × B6–.

BtSV tissue localisation in LE+ female body region and tissues

Kruskal-Wallis rank sum test

Kruskal-Wallis chi-squared = 1.395, df = 2, p-value = 0.4978

Dunn (1964) Kruskal-Wallis multiple comparison
p-values adjusted with the Benjamini-Hochberg method.

	Comparison	Z	P.unadj	P.adj
1	Gut - Head	-1.1667262	0.2433210	0.7299629
2	Gut - Ovaries	-0.4242641	0.6713732	0.6713732
3	Head - Ovaries	0.7424621	0.4578074	0.6867111

BtSV tissue localisation in LE+ male body region and tissues

Kruskal-Wallis rank sum test

Kruskal-Wallis chi-squared = 0.26, df = 2, p-value = 0.8781

Dunn (1964) Kruskal-Wallis multiple comparison
p-values adjusted with the Benjamini-Hochberg method.

	Comparison	Z	P.unadj	P.adj
1	Gut - Head	-0.1414214	0.8875371	0.8875371
2	Gut - Testes	-0.4949747	0.6206179	1.0000000
3	Head - Testes	-0.3535534	0.7236736	1.0000000

BtSV tissue localisation in female offspring of LE+ × B6– family 5

Kruskal-Wallis rank sum test

Kruskal-Wallis chi-squared = 1.8846, df = 2, p-value = 0.3897

Dunn (1964) Kruskal-Wallis multiple comparison
p-values adjusted with the Benjamini-Hochberg method.

	Comparison	Z	P.unadj	P.adj
1	Gut - Head	-0.1961161	0.8445193	0.8445193
2	Gut - Ovaries	-1.2747549	0.2023960	0.6071880
3	Head - Ovaries	-1.0786387	0.2807488	0.4211232

BtSV tissue localisation in female offspring of LE+ × B6– family 6

Kruskal-Wallis rank sum test

Kruskal-Wallis chi-squared = 9.8462, df = 2, p-value = 0.007277

Dunn (1964) Kruskal-Wallis multiple comparison
p-values adjusted with the Benjamini-Hochberg method.

	Comparison	Z	P.unadj	P.adj
1	Gut - Head	-1.568929	0.116664465	0.116664465
2	Gut - Ovaries	-3.137858	0.001701872	0.005105616
3	Head - Ovaries	-1.568929	0.116664465	0.174996697

BtSV tissue localisation in male offspring of LE+ × B6– family 5

Kruskal-Wallis rank sum test

Kruskal-Wallis chi-squared = 6.2692, df = 2, p-value = 0.04352

Dunn (1964) Kruskal-Wallis multiple comparison
p-values adjusted with the Benjamini-Hochberg method.

	Comparison	Z	P.unadj	P.adj
1	Gut - Head	-1.6669871	0.09551696	0.14327544
2	Gut - Testes	0.7844645	0.43276758	0.43276758
3	Head - Testes	2.4514517	0.01422813	0.04268439

BtSV tissue localisation in male offspring of LE+ × B6– family 6

Kruskal-Wallis rank sum test

Kruskal-Wallis chi-squared = 7.7308, df = 2, p-value = 0.02095

Dunn (1964) Kruskal-Wallis multiple comparison
p-values adjusted with the Benjamini-Hochberg method.

	Comparison	Z	P.unadj	P.adj
1	Gut - Head	-2.6475678	0.00810731	0.02432193
2	Gut - Testes	-0.5883484	0.55629846	0.55629846
3	Head - Testes	2.0592194	0.03947322	0.05920984

BtSV tissue localisation in female offspring of (LE+ × B6–) × B6– family 1

Kruskal-Wallis rank sum test

Kruskal-Wallis chi-squared = 4.6222, df = 2, p-value = 0.09915

Dunn (1964) Kruskal-Wallis multiple comparison
p-values adjusted with the Benjamini-Hochberg method.

	Comparison	Z	P.unadj	P.adj
1	Gut - Head	1.4907120	0.13603713	0.2040557
2	Gut - Ovaries	2.0869968	0.03688843	0.1106653
3	Head - Ovaries	0.5962848	0.55098499	0.5509850

BtSV tissue localisation in female offspring of (LE+ × B6–) × B6– family 2

Kruskal-Wallis rank sum test

Kruskal-Wallis chi-squared = 4.5714, df = 2, p-value = 0.1017

Dunn (1964) Kruskal-Wallis multiple comparison
p-values adjusted with the Benjamini-Hochberg method.

	Comparison	Z	P.unadj	P.adj
1	Gut - Head	1.069045	0.28504941	0.28504941
2	Gut - Ovaries	2.138090	0.03250944	0.09752833
3	Head - Ovaries	1.069045	0.28504941	0.42757411

BtSV tissue localisation in male offspring of B6– × (LE+ × B6–) family 1

Kruskal-Wallis rank sum test

Kruskal-Wallis chi-squared = 5.6, df = 2, p-value = 0.06081

Dunn (1964) Kruskal-Wallis multiple comparison
p-values adjusted with the Benjamini-Hochberg method.

	Comparison	Z	P.unadj	P.adj
1	Gut - Head	0.4472136	0.65472085	0.65472085
2	Gut - Testes	2.2360680	0.02534732	0.07604196
3	Head - Testes	1.7888544	0.07363827	0.11045741

BtSV tissue localisation in male offspring of B6– × (LE+ × B6–) family 2

Kruskal-Wallis rank sum test

Kruskal-Wallis chi-squared = 3.5, df = 2, p-value = 0.1738

Dunn (1964) Kruskal-Wallis multiple comparison
p-values adjusted with the Benjamini-Hochberg method.

	Comparison	Z	P.unadj	P.adj
1	Gut - Head	-0.09805807	0.92188618	0.9218862
2	Gut - Testes	1.56892908	0.11666446	0.1749967
3	Head - Testes	1.66698715	0.09551696	0.2865509

BtSV tissue localisation in female offspring of B6– × (LE+ × B6–) family 1

Kruskal-Wallis rank sum test

Kruskal-Wallis chi-squared = 6.5, df = 2, p-value = 0.03877

Dunn (1964) Kruskal-Wallis multiple comparison
p-values adjusted with the Benjamini-Hochberg method.

	Comparison	Z	P.unadj	P.adj
1	Gut - Head	0.09805807	0.92188618	0.92188618
2	Gut - Ovaries	2.25533555	0.02411227	0.07233682
3	Head - Ovaries	2.15727749	0.03098405	0.04647608

BtSV tissue localisation in female offspring of B6– × (LE+ × B6–) family 2

Kruskal-Wallis rank sum test

Kruskal-Wallis chi-squared = 7.3846, df = 2, p-value = 0.02491

Dunn (1964) Kruskal-Wallis multiple comparison
p-values adjusted with the Benjamini-Hochberg method.

	Comparison	Z	P.unadj	P.adj
1	Gut - Head	0.000000	1.00000000	1.00000000
2	Gut - Ovaries	2.353394	0.01860293	0.02790439
3	Head - Ovaries	2.353394	0.01860293	0.05580879

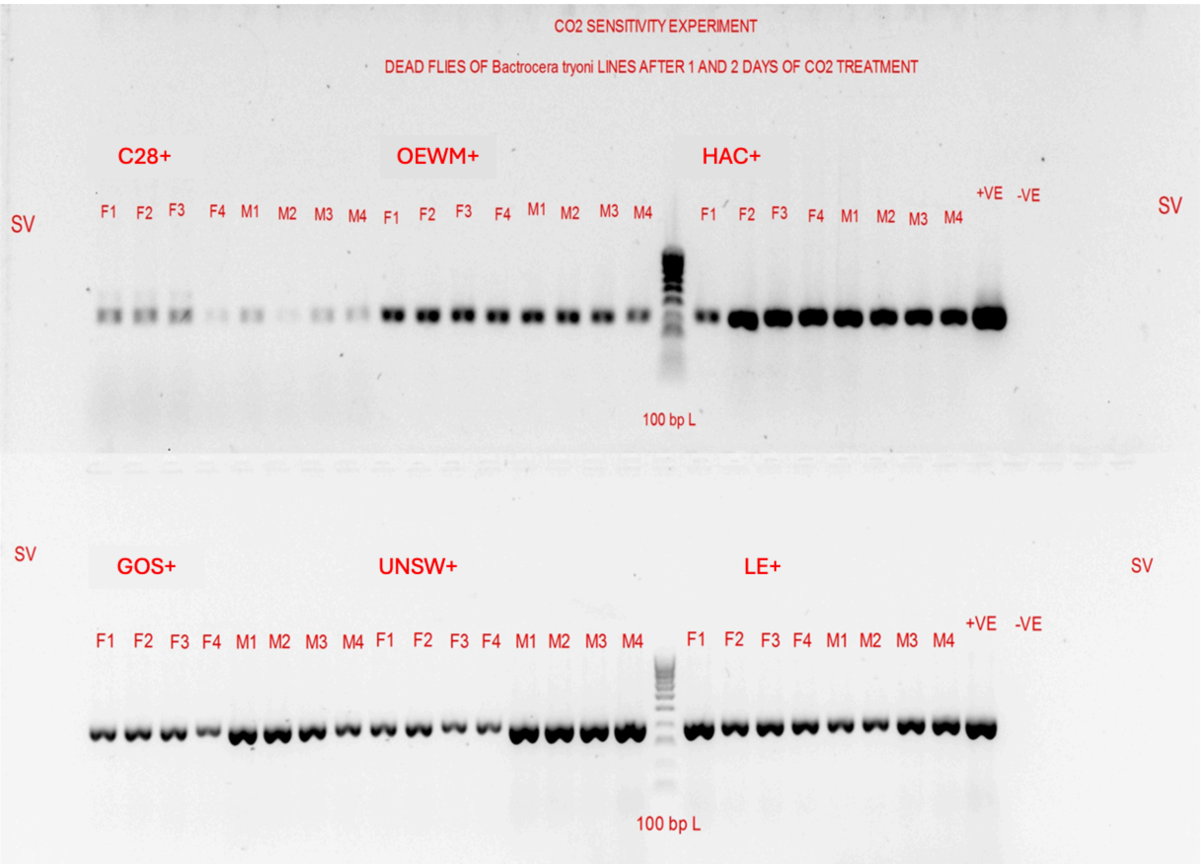

SI 8. Standard RT-PCR for the RdRp gene of BtSV in flies that died 1 and 2 days after CO_2 treatment of 12 *Bactrocera tryoni* lines.

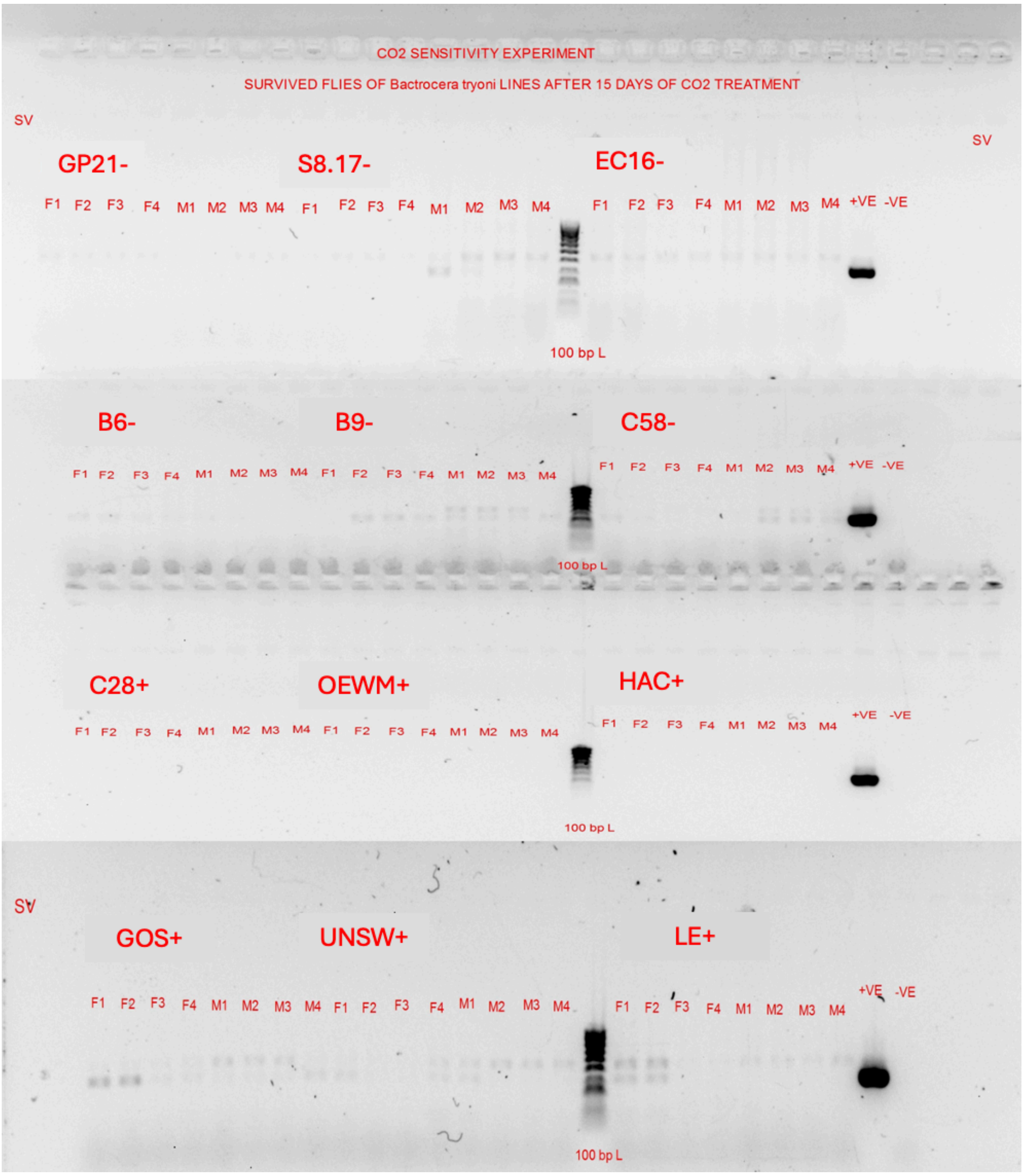

SI 9. Standard RT-PCR for the RdRp gene of BtSV in flies that were recovered alive 15 days after CO_2 treatment of 12 *Bactrocera tryoni* lines.

SI 10. Cox proportional hazards model survival analysis of BtSV-infected and uninfected populations

```
coxph(formula = Surv(Day, Dead > 0) ~ Fly_virus_status, data = data,
    cluster = Population)

  n= 192, number of events= 120
```

	coef	exp(coef)	se(coef)	robust se	z
Sigmavirus positive	0.6363	1.8894	0.1914	0.1794	3.547

Pr(>|z|)
Sigmavirus positive 0.00039 ****

Signif. codes: 0 ‘***’ 0.001 ‘**’ 0.01 ‘*’ 0.05 ‘.’ 0.1 ‘ ’ 1

	exp(coef)	exp(-coef)	lower.95	upper.95
Sigmavirus positive	1.889	0.5293	1.329	2.686

Concordance= 0.638	(se = 0.044)	
Likelihood ratio test	= 11.6 on 1 df,	p=7e-04
Wald test	= 12.58 on 1 df,	p=4e-04
Score (logrank) test	= 11.43 on 1 df,	p=7e-04,Robust = 7.5 p=0.006

(Note: the likelihood ratio and score tests assume independence of observations within a cluster, the Wald and robust score tests do not).

confidence interval	2.5 %	97.5 %
Sigmavirus positive	1.329307	2.685597

> **pval 0.0003901423**
